# Depression and Apathy After Transient Ischemic Attack or Minor Stroke: Prevalence, Evolution and Predictors

**DOI:** 10.1038/s41598-019-52721-5

**Published:** 2019-11-07

**Authors:** Anna Carnes-Vendrell, Joan Deus, Jessica Molina-Seguin, Josep Pifarré, Francisco Purroy

**Affiliations:** 1 Clinical Psychologist and Neuropsychologist. Neurology department, Hospital Universitari de Santa Maria, Lleida, Spain; 2 Clinical Neuroscience Group of the Biomedical Research Institute of Lleida (IRBLleida), Universitat de Lleida, Lleida, Spain; 3 University of Barcelona, Spain. MRI Research Unit, Department of Radiology, Hospital del Mar, Barcelona, Spain; 4 Department of Clinical and Health Psychology, Faculty of Psychology at the Autonomous University of Barcelona, Barcelona, Spain; 5 Neurology Service Hospital Universitari Mutua de Terrassa. Clinical Neuroscience Group of the Biomedical Research Institute of Lleida (IRBLleida). Universitat de Lleida, Lleida, Spain; 6 Psychiatrist. Biomedical Research Institute of Lleida (IRBLleida) Universitat de Lleida, Lleida, Spain; 7 Stroke Unit. Hospital Universitari Arnau de Vilanova, Lleida, Spain

**Keywords:** Cognitive ageing, Neurology

## Abstract

Few previous studies have focused on affective impairment after transient ischemic attack (TIA) and/or minor stroke. The aim was to establish the prevalence, evolution and predictors of post-stroke depression (PSD) and post-stroke apathy (PSA) over a 12-month follow-up period. We prospectively included TIA and minor stroke patients (NIHSS ≤4) who had undergone magnetic resonance imaging <7 days. PSD was diagnosed according to DSM-5 criteria and PSA was defined based on an Apathy Evaluation Scale (AES-C) score of ≥37. Clinical and neuroimaging variables (presence and patterns of lesion, cerebral bleeds and white matter disease) were analysed in order to find potential predictors for PSD and PSA. Follow-up was performed at 10 days and after 2, 6, 9 and 12 months. 82 patients were included (mean 66.4 [standard deviation11.0] years) of whom 70 completed the follow-up. At 10 days, 36 (43.9%) and 28 (34.1%) patients respectively were diagnosed with PSD and PSA. At 12 months, 25 of 70 (35.7%) patients still had PSA, but only 6 of 70 (8.6%) had PSD. Beck Depression Inventory-II score, mini mental state examination (MMSE) and a previous history of depression or anxiety were predictors for PSD. While MMSE score, The Montgomery Asberg Depression Rating Scale and having previously suffered a stroke were also risk factors for PSA. Acute basal ganglia lesion and periventricular leukoaraiosis were associated with PSA while deep leukorariosis with PSD. Despite the presence of few or only transient symptoms, PSD and PSA frequent appear early after TIA and minor stroke. Unlike PSD, apathy tends to persist during follow-up.

## Introduction

The development of depressive symptoms is the most frequent affective complication after a stroke. Post-stroke depression (PSD) has been widely studied and has been estimated to affect between 29% and 33% of patients^1,2^. PSD is associated with a worse prognosis, worse functional recovery, greater difficulties for social reintegration, poorer quality of life and an increased risk of stroke recurrence^3–5^. It has also been reported that patients with PSD have a three to four times higher risk of mortality than non-depressed patients^6^.

Although apathy and depression may appear together, and the former may be a symptom or expression of the latter, it has been shown that apathy can also occur as an independent symptom of depression^7^. Unlike PSD, post-stroke apathy (PSA) has not hitherto received much attention, even though it affects one in three stroke patients, with only 40% of cases being concomitant with depression^8–10^. It has also been shown that PSA is consistently associated with a worse level of functional recovery, poorer overall health and poorer quality of life^9,11,12^.

In both cases, a number of predictors have been described. However, most of the studies conducted to date have mainly focused on the established stroke population, excluding patients with transient ischemic attack (TIA) and/or minor stroke. Despite minimum or complete resolution of the neurological symptoms, these patients remain vulnerable to these neuro-psychiatric complications^13^.

Although magnetic resonance imaging variables are essential not only for the diagnosis of TIA, based on its tissular definition^14^, but also for the prediction of stroke recurrence after TIA or minor stroke^15^, little information is currently available about neuroimaging data and the development of PSD and PSA in these patients^16,17^. Diffusion-weighted imaging could detect signs of acute ischemia in 30% of TIA patients. Besides their transient symptoms, these lesions could also increase the risk of cognitive and affective impairment. A relationship has been also been described between the location of infarcts, the presence of white matter lesions^18^ and cerebral microbleeds^8,19^, and cognitive and affective impairment. Our aim was therefore to determine the prevalence, predictors and evolution of symptoms of post-stroke depression and post-stroke apathy in TIA and minor stroke patients during the first post-stroke year.

## Methods

### Patients and study design

We developed our observational, prospective and longitudinal studies according to STROBE guidelines^20^. Baseline (10 days after the onset of symptoms) and follow-up visits were performed at 2, 6, 9 and 12 months. The study included TIA and minor stroke patients aged from 18 to 85 years old who had been consecutively admitted to the Hospital Universitari Arnau de Vilanova (HUAV) between January and December 2015. The Scientific Ethics Committee of the HUAV approved both the study and the consent procedure. We obtained written informed consent from all the patients involved. The whole process was performed in accordance with the relevant guidelines and regulations.

TIA was defined as a rapidly evolving focal neurological deficit with only a vascular cause and a duration of less than 24 h. Minor stroke was defined as an ischemic stroke with a score of ≤4 on the NIHSS^21^. All the patients were submitted to neurological examination by a stroke neurologist. We excluded patients with modified Rankin Scale Scores (mRS) of >3, mild cognitive impairment (MCI) or dementia diagnosed before the stroke event, life expectancies of less than 1 year, language barriers and/or illiteracy.

Risk factor profiles, clinical and sociodemographic characteristics, and severity (National Institutes of Health Stroke Scale, NIHSS score^22^) of stroke were all recorded. Patients were classified etiologically according to TOAST (Trial of Org 10172 in Acute Stroke Treatment^22^) criteria as due to large-artery occlusive disease, small-vessel disease, cardioembolism, other cause and undetermined cause. For strokes of undetermined origin, no cause was found despite an extensive evaluation or a most likely cause could not be determined because more than one plausible cause was found.

### Neuroimaging evaluation

An MRI was acquired using a 1.5-T whole-body imager system with 24-mT/m gradient strength, 300-ms rise time, and an echo-planar–capable receiver equipped with a gradient overdrive (Philips Intera 1.5 T, MRI scanner). A neuroradiologist, who was blind to the clinical features, established the presence and location of any diffusion weighted imaging abnormalities. Lesion locations of acute infarcts were classified as frontal lobe, temporal lobe, parietal lobe, occipital lobe, subcortical white matter, basal ganglia, thalamus, brain stem or cerebellum. As previously described^15^, patterns of lesion were defined as: MRI normality, scattered pearls in one arterial territory (SPOT), multiple vascular territories, a single cortical lesion in one vascular territory, and a subcortical pattern. Acute ischemic lesions were also classified as lesions affecting the anterior cerebral artery territory, cortical medial cerebral artery (MCA) territory, deep medial cerebral artery territory, artery penetrating territory, posterior cerebral artery territory and brain stem or cerebellum territory.

OsiriX V.4.0 imaging software was used to calculate the total volume of the diffusion weighted imaging abnormality in cubic centimetres. Leukoaraiosis was categorized into subcortical and periventricular leukoaraiosis. Subcortical leukoaraiosis denoted white-matter hyperintensities in the centrum semiovale and the corona radiata (0: absent, 1: punctate foci, 2: initial confluence of foci 3: large confluent areas). Periventricular leukoaraiosis indicated white-matter hyperintensities exclusively located around the lateral ventricles (0: absent, 1: caps or pencil lining, 2: smooth halo, 3: irregular periventricular hyperintensity extending into deep white matter). Cerebral microbleeds were defined as multiple ovoid foci with a marked loss of signal intensity on T2*-weighted, gradient-recalled echo MRI^23^. Cerebral microbleeds were classified into lobar (cortex and subcortical white matter) and deep (basal ganglia, internal and external capsules, thalamus and posterior fossa). In all cases, MRI was performed within 1 week of hospital admission (3.7 [SD 1.5] days).

### Depression and apathy assessment

A mental health professional made the diagnosis of PSD based on information reported in two assessment questionnaires: the Beck Depression Inventory (BDI-2)^24^ and Montgomery-Asberg Depression Rating Scale (MADRS)^25^. The BDI-2 is a 21-question multiple-choice self-report inventory and is one of the most widely used psychometric tests for measuring the severity of depression. The MADRS is a 10-item diagnostic questionnaire which is also used to measure the severity of depressive episodes. Both instruments have been extensively used to study the stroke population and to determine their sensitivity and specificity; both have also provided satisfactory results that demonstrate their usefulness in stroke patients^26–29^. Patients diagnosed with PSD had to meet the DMS-5 criteria.

Those patients who were diagnosed with depression were referred to the psychiatrist service. The same psychiatrist evaluated the patients during the following week and before any medication was prescribed. He decided what would constitute the best medical treatments (serotonin reuptake inhibitors) based on current guidelines^30,31^.

PSA was assessed using the clinician version of the Apathy Evaluation Scale (AES-C)^32^, which is based on the current functioning of patients. This assessment contains 18 questions and is administered as a semi-structured interview. As in previous studies, we used a cut-off score of 37 on the AES-C to identify patients with PSA^8,33^.

### Cognitive, functional and quality of life variables

Two screening tests were administered: the Mini Mental State Examination (MMSE) and the Montreal Cognitive Assessment (MoCA), to evaluate global deterioration at the baseline level.

Functionality was evaluated using the Barthel Index (BI)^34^ and the modified Rankin Scale (mRS)^35^. The BI scale consists of 10 items which are used to evaluate the basic activities of daily life. A high score indicates a greater level of independence for the basic activities of daily living. The mRS allowed us to assess each patient’s degree of functional disability on a 7-level scale of severity (scored from 0 to 6). A low score indicates good functionality and autonomy.

Quality of life was determined using the Quality of Life Scale for Stroke (ECVI-38)^36,37^. This is a self-administered, 38-item scale that evaluates the degree of difficulty that people encounter in different areas. High scores indicate greater difficulties and, therefore, a worse perceived quality of life.

### Statistical analysis

Data were described as frequencies and percentages for qualitative variables, mean and standard deviations (SD) for normally distributed quantitative variables, and median and interquartile ranges (IR) for non-normally distributed variables. Bivariate analysis of baseline and 12-month main outcomes: PSD (yes vs. no) and PSA (AES-C ≥ 37 vs. AES-C < 37) with other study variables were performed. Qualitative variables were analysed using the Pearson’s Chi-square test, and quantitative variables using the Student’s t-test or the Mann-Whitney U test. The longitudinal trajectories of affectivity and apathy variables (BDI-II, MADRS, AES-C) were also described by non-parametric local polynomial regression (loess smoothing). Finally, we fitted multivariable logistic regression models for each study outcome, both baseline and 12-months. Variables showing p < 0.1 on univariate testing were included in different logistic-regression models that used the forward stepwise method to identify predictors of PSD and PSA at baseline and at 12-months. Model A only included the psychometric instrument scores; Model B scores from psychometric instruments, except BDI-II and MADRS (model B); and Model C is like model B but adding the rest of the sociodemographic and baseline clinical and neuroimaging variables. Model calibration was assessed with the Hosmer-Lemeshow goodness-of-fit test and the area under the ROC curve was obtained as a measure of the ability to discriminate. All the tests were bilateral and run at a significance level of 5%. All the analyses were carried out using the statistical software R 3.5.1.

## Results

### Sample characteristics

**Figure 1:**
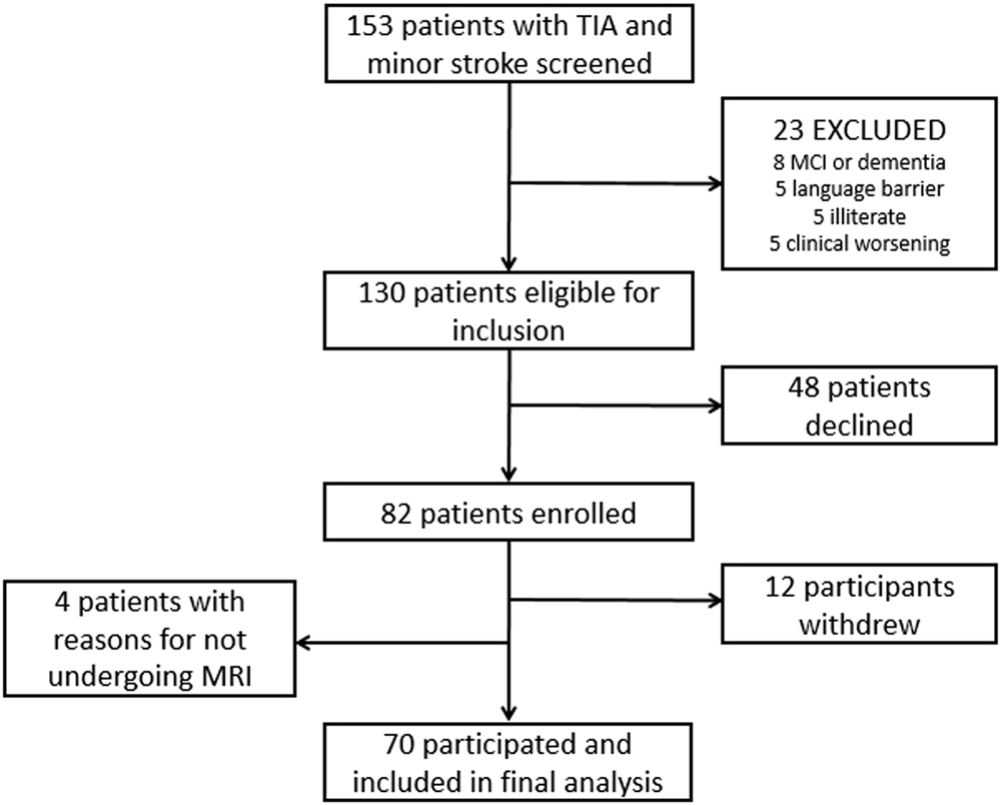
Flow-chart.

From the initial 130 eligible patients, 82 patients were included and 70 completed the follow-up (Fig. 1 – flow chart). Of the 82 patients, 42 suffered a minor stroke and 40 suffered a TIA. 48 of the 130 eligible patients declined to participate in the study, mainly because they refused to go to the hospital to do the visits. There were no significant differences between the sociodemographic characteristics of the patients who participated in the study and those who did not. 12 patients dropped out, basically for the same reasons as previous stated, during the first or second follow-up visit stage.

The mean age of the patients included in the study was 66.4 (SD 11.0) years old and 72% of them were male (Table 1). Hypertension was the main risk factor in 48 (68.3%) of the cases. Based on the etiological classification of the TOAST, 40.2% were classified as indeterminate stroke patients.

**Table 1:** Sociodemographic and clinical variables related to post-stroke depression and post-stroke apathy (baseline data).

	All	Post-stroke depression (PSD)			Post-stroke apathy (PSA)		
		Non PSD patients N = 53	PSD patients N = 29	*p*	Non PSD patients N = 46	PSA patients N = 36	*p*
**Sociodemographic and clinical variables**							
**Age, mean (standard deviation)**	66.4 (11.0)	66.6 (11.0)	65.8 (11.1)	0.751	66.8 (11.1)	65.8 (10.9)	0.691
**Gender:**				0.483			0.075
Male	59 (72.0%)	40 (75.5%)	19 (65.5%)		29 (63.0%)	30 (83.3%)	
Female	23 (28.0%)	13 (24.5%)	10 (34.5%)		17 (37.0%)	6 (16.7%)	
**Family status:**				0.296			0.541
Married	63 (76.8%)	37 (69.8%)	26 (89.7%)		33 (71.7%)	30 (83.3%)	
Widower	11 (13.4%)	9 (17.0%)	2 (6.9%)		7 (15.2%)	4 (11.1%)	
Divorced/separated	6 (7.3%)	5 (9.4%)	1 (3.4%)		5 (10.9%)	1 (2.8%)	
Single	2 (2.4%)	2 (3.8%)	0 (0.0%)		1 (2.2%)	1 (2.8%)	
**Employment status:**				**0.029**			0.058
Employee	22 (26.8%)	17 (32.1%)	5 (17.2%)		14 (30.4%)	8 (22.2%)	
Unemployed	4 (4.9%)	1 (1.9%)	3 (10.3%)		2 (4.3%)	2 (5.6%)	
Retired	51 (62.2%)	34 (64.2%)	17 (58.6%)		30 (65.2%)	21 (58.3%)	
Inability to work	5 (6.1%)	1 (1.9%)	4 (13.8%)		0 (0.0%)	5 (13.9%)	
**Level of education:**				0.240			0.818
Primary education	57 (69.5%)	34 (64.2%)	23 (79.3%)		31 (67.4%)	26 (72.2%)	
Secondary education or more	25 (30.5%)	19 (35.8%)	6 (20.7%)		15 (32.6%)	10 (27.8%)	
**Occasional alcohol consumption**	38 (46.3%)	27 (50.9%)	11 (37.9%)	0.369	13 (28.3%)	25 (69.4%)	**<0.001**
**Smoking:**				0.267			0.683
Non-smoker	31 (37.8%)	18 (34.0%)	13 (44.8%)		19 (41.3%)	12 (33.3%)	
Ex-smoking	28 (34.1%)	17 (32.1%)	11 (37.9%)		14 (30.4%)	14 (38.9%)	
Smoking	23 (28.0%)	18 (34.0%)	5 (17.2%)		13 (28.3%)	10 (27.8%)	
**Diabetes mellitus**	30 (36.6%)	14 (26.4%)	16 (55.2%)	**0.019**	16 (34.8%)	14 (38.9%)	0.879
**Hypertension**	56 (68.3%)	37 (69.8%)	19 (65.5%)	0.880	32 (69.6%)	24 (66.7%)	0.967
**Hypercholesterolemia**	37 (45.1%)	23 (43.4%)	14 (48.3%)	0.847	19 (41.3%)	18 (50.0%)	0.574
**Atrial fibrillation**	5 (6.1%)	3 (5.7%)	2 (6.9%)	1.000	3 (6.5%)	2 (5.6%)	0.999
**Previous psychiatric disease**	28 (34.1%)	12 (22.6%)	16 (55.2%)	**0.006**	13 (28.3%)	15 (41.7%)	0.300
**Family history:**				0.331			0.128
Neurological disease	30 (36.6%)	18 (34.0%)	12 (41.4%)		20 (43.5%)	10 (27.8%)	
Psychiatric disease	10 (12.2%)	5 (9.4%)	5 (17.2%)		3 (6.5%)	7 (19.4%)	
**NIHSS score at admission, median (interquartile interval)**	1.0 (0.0–2.8)	1.0 [0.0–2.0]	2.0 [0.0–3.0]	0.063	1.0 (0.0–2.0)	2.0 (0.0–3.0)	0.092
**Previous stroke**	14 (17.1%)	9 (17%)	5 (17.2%)	0,999	4 (8.7%)	10 (27.8)%	**0.047**
**Etiology of stroke:**				0.516			0.464
Large artery atherosclerotic	18 (22.0%)	10 (18.9%)	8 (27.6%)		9 (19.6%)	9 (25.0%)	
Cardioembolism	13 (15.9%)	9 (17.0%)	4 (13.8%)		6 (13.0%)	7 (19.4%)	
Lacunar	18 (22.0%)	14 (26.4%)	4 (13.8%)		9 (19.6%)	9 (25.0%)	
Undetermined	33 (40.2%)	20 (37.7%)	13 (44.8%)		22 (47.8%)	11 (30.6%)	

### Neuroimaging data

The neuroimaging features are displayed in online Supplementary Table 1 and in the online Supplementary Information. 44 (59.5%) of the patients had positive diffusion-weighted imaging. Most of the acute ischemic lesions affected the cortical MCA territory (28.4%).

### Description and evolution of depression and post-stroke apathy

29 (35.36%) patients were initially diagnosed as suffering PSD. The median scores for the BDI-II and the MADRS scores were 5.00 (IR 3.0–9.0) and 4.0 (IR 2.0–9.0), respectively. 36 (43.90%) patients had PSA at baseline. The median AES-C scale score was 34.0 (IR 28.0–42.8). 16 (19.5%) of the patients suffered from PSD and PSA. Table 2 shows the proportion of patients independently suffering from depression and apathy and also those who suffered them concomitantly, both at baseline and at the 12-month follow-up.

**Table 2:** Proportion of patients diagnosed with post-stroke depression, post-stroke apathy, or both.

	Baseline N = 82	12-month follow-up N = 70
Post-stroke depression	13 (15.8%)	1 (2.2%)
○ TIA	4 (10%)	
○ Minor stroke	9 (21.4%)	1 (2.5%)
Concomitant post-stroke depression and apathy	16 (19.5%)	7 (10%)
○ TIA	8 (20.0%)	2 (8%)
○ Minor stroke	8 (19.0%)	5 (20%)
Post-stroke apathy	20 (19.5%)	18 (25.7%)
○ TIA	6 (15%)	4 (11.1%)
○ Minor stroke	14 (33.3%)	12 (35.3%)

**Figure 2:**
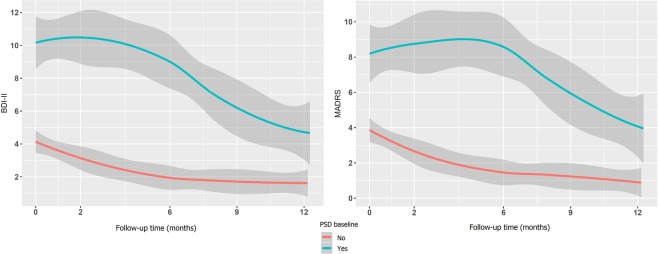
Evolution of post-stroke depression. Figure shows that there was a decrease in the level of depression, which is shown by the BDI-II and MADRS scales. In baseline PSD patients (blue line), both scores decreased at 12 months (the red line indicates healthy patients, without baseline PSD).

**Figure 3:**
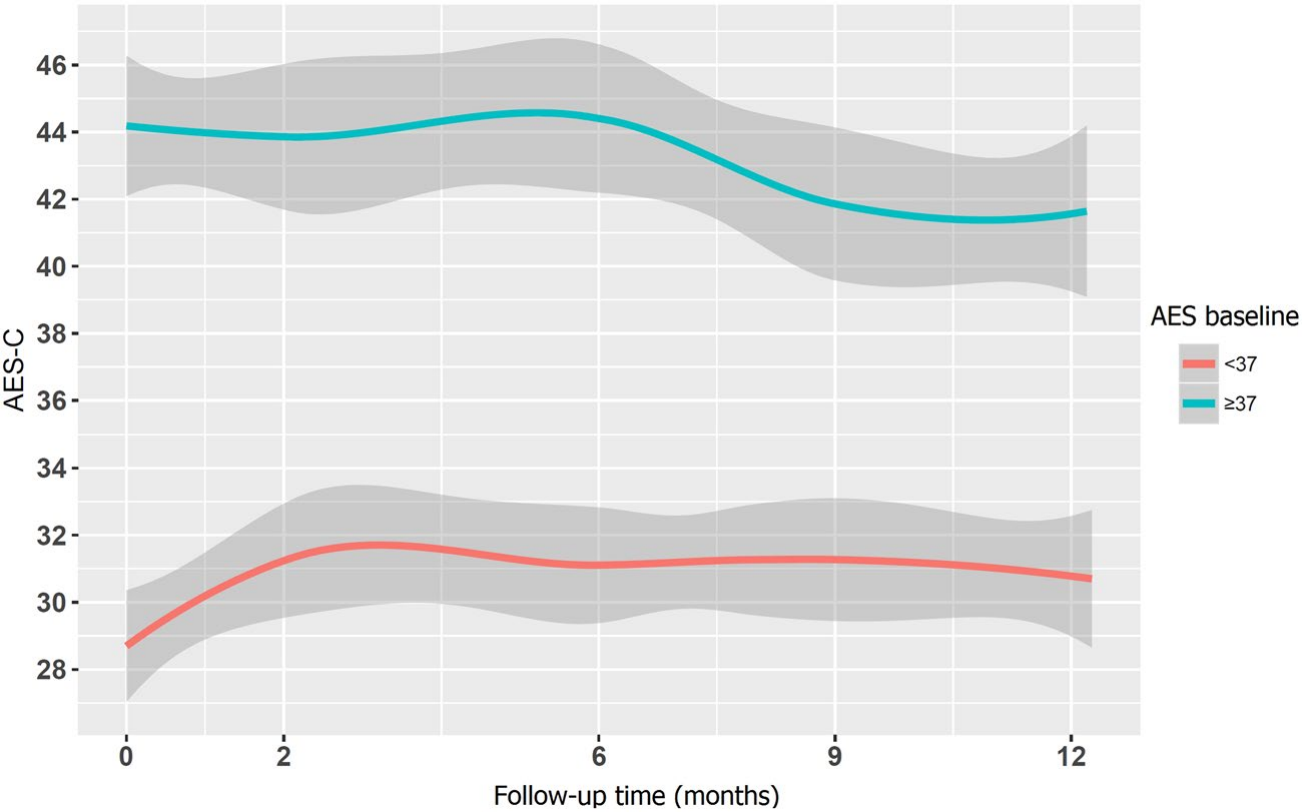
Evolution of post-stroke apathy. Figure shows how apathy remained stable during the first year post-stroke; the level of apathy measured by the AES-C score did not decrease (blue line). The red line indicates patients without post-stroke apathy at baseline (<37 scores at AES-C).

After the first 12 months of follow-up, the proportion of patients with PSD decreased significantly to 11.42%. In contrast, the proportion of patients with PSA remained similar to the baseline value (Figs 2 and 3). All of the patients who had post-stroke depression and post-stroke apathy at the 12-month follow-up were initially diagnosed at baseline.

### Variables related to post-stroke depression

According to the bivariate analysis, at baseline and at 12 months (Table 2 and Supplementary Tables 2 and 3), PSD patients who exhibited cases of diabetes mellitus were more likely to be unemployed (p = 0.029) and to have personal psychiatric histories than non-PSD patients. The latter exhibited higher levels of apathy, reported worse scores for quality of life (p = 0.001) and worse levels of cognitive performance (according to the MMSE) than non-PSD patients. BDI-II, MADRS and NIHSS scores were only related to PSD at baseline. No significant differences in neuroimaging variables were observed between patients with and without PSD at baseline, either between patients who had suffered TIA or minor stroke. Fazekas hyperintensities in the deep white matter substance score was related to PSD at 12 months.

In the different multivariate logistic regression models (Table 3), BDI-II MMSE, personal psychiatric history, ECVI-38, NIHSS score and diabetes mellitus were all identified as independent predictors of PSD at baseline. AES-C was the only predictor of PSD at 12 months.

**Table 3:** Multivariate analysis for post-stroke depression after TIA and/or minor stroke.

	Baseline			12 m follow-up
	Model A	Model B	Model C	Model A
	OR (CI 95%); p value	OR (CI 95%); p value	OR (CI 95%); p value	OR (CI 95%); p value
BDI-II	1.35 (1.19–1.58); <0.001	—	—	—
ECVI-38	—	1.09 (1.03–1.16); 0.006	—	—
MMSE	—	0.78 (0.63–0.94); 0.014	—	—
AES-C	—	—	—	1.53 (1.03–1.35); 0.026
Personal psychiatric history	—	—	6.25 (1.98–22.92); 0.003	
NIHSS	—	—	6.6 (2.12–24.55); 0.002	—
DM	—	—	4.04 (1.34–13.2); 0.015	—
H-L test, p-value	0.82	0.69	0.94	0.40
AUC ROC (CI 95%)	0.82 (0.72–0.92)	0.76 (0.65–0.87)	0.79 (0.69–0.89)	0.79 (0.57–1.03)

Abbreviations: BDI-II: Beck Depression Inventory; ECVI-38: Stroke Quality of Life Scale; MMSE: Mini-Mental State Examination; AES-C: Apathy Evaluation Scale; NIHSS: National Institute of Health Stroke Scale; DM: Diabetes Mellitus. H-L: Hosmer-Lemeshow; AUC: area under the curve.

Model A: multivariate analysis including only scores from psychometric instruments.

Model B: multivariate analysis including only scores from psychometric instruments, except the BDI-II and MADRS.

Model C: multivariate analysis including sociodemographic, clinical, neuroimaging and psychometric instruments (excluding BDI-II and MADRS).

### Variables related to post-stroke apathy

According to the bivariate analysis (Supplementary Tables 2 and 3), employment status, NIHSS score, AES-C, BDI-II, MADRS and ECVI-38 scores were all related to PSA, both at baseline and at 12 months. The occasional consumption of alcohol, previous stroke, the presence of acute lesion in the basal ganglia and the Fazekas periventricular score were all associated with basal PSA. Cognitive impairment determined by MMSE and primary studies were related to PSA at 12 months. There were no significant differences between patients with and without PSA in relation to their diagnosis (TIA or minor stroke).

In the different logistic regression models (Table 4), MADRS, previous stroke and occasional alcohol consumption were predictors of PSA at baseline. MMSE and AES-C were identified as predictors of PSA at 12 months (Table 3). Interestingly, basal PSD was not only a predictor for PSA at baseline but also for persistent PSA. Finally, secondary or further education was inversely related to PSA at 12 months.

**Table 4:** Multivariate analysis for post-stroke apathy after TIA and/or minor stroke.

	Baseline		12 m follow-up	
	Model A	Model C	Model A	Model C
	OR (CI 95%); p value	OR (CI 95%); p value	OR (CI 95%); p value	OR (CI 95%); p value
MADRS	1.18 (1.06–1.32); 0.0034	—	—	—
PSD	—	3.43 (1.16–11.3); 0.0321	—	4.34 (1.08–19.8); 0.0433
MMSE	—	—	0.78 (0.58–0.99); 0.0587	—
AES-C	—	—	1.25 (1.14–1.42); <0.0001	1.25 (1.14–1.42); <0.0001
Secondary education or more	—	—	—	0.19 (0.03–0.93); 0.0559
Occasional alcohol consumption	—	8.42 (2.94–27.8); 0.0002	—	—
Previous stroke	—	5.0 (1.31–23.03); 0.0249	—	—
H-L test, p-value	0.97	0.43	0.49	0.17
AUC ROC (CI 95%)	0.69 (0.58–0.81)	0.79 (0.69–0.89)	0.87 (0.78–0.97)	0.88 (0.79–0.97)

Abbreviations: MADRS: Montgomery-Asberg Depression Rating Scale; PSD: Post-Stroke Depression; MMSE: Mini-Mental State Examination; AES-C: Apathy Evaluation Scale. H-L: Hosmer-Lemeshow; AUC: area under the curve.

Model A: multivariate analysis including only scores from psychometric instruments.

Model C: multivariate analysis including sociodemographic, clinical, neuroimaging and psychometric instruments (excluding BDI-II and MADRS).

## Discussion

Few previous studies have focused on the development and evolution of PSD and PSA after TIA and/or minor stroke. In our prospective study, most of the PSD patients improved over time. In contrast, PSA persisted throughout the follow-up period. Two hypotheses about this finding can be made. Firstly, it is more common to evaluate depression and to include apathy symptoms within the depressive syndrome. Secondly, it may be that patients and their families do not complain about apathy as much as they do about depression. These two factors can mean that PSA often goes unnoticed by clinicians^38^. In such cases, no specific treatment will be given to it and this would perhaps explain the persistence of apathy over time. Furthermore, in those cases in which it is treated (not those in our study), it has been noticed that apathy is more resistant to pharmacological treatments than depression^31,38^.

The two affective symptoms were apparently related. Basal apathy was associated with persistent depression and basal depression with basal apathy and persistent apathy. This relationship had also been previously described. It has been previously reported that depression constitutes a risk factor for the appearance of apathy as an independent syndrome^9,10,39–41^. In our sample, 19.6% of the patients with PSA also had PSD. We did not identify any patients with PSA at 12 months who did not also have PSA at baseline. PSA has not traditionally received as much attention as PSD. However, although these two neuropsychiatric complications may be confused, scientific interest in determining the predictive factors and neuroanatomic causes underlying them has increased in recent years, and especially in patients with Parkinson’s disease. Our proportion of PSA and PSD is lower than in other studies^9,42,43^. The heterogeneity of reported rates of PSA and PSD could be explained by the differences in the evalutaion methods used. In our study, thediagnosis was rigorous and required not only a determined score in a specific questionnaire but also an evaluation by a mental health professional. Although we did not observe a relationship between diffusion-weighted imaging abnormalities and affective complications, the proportions of PSA and PSD were as high as those determined in previous articles that included ischemic stroke patients. In this sense, Turner *et al*. previously observed that the risk of developing fatigue, psychological impairment and cognitive impairment in TIA patients was significantly higher than in controls^44^. Interestingly, we found that both affective complications had specific predictors; for example, low MMSE scores predicted PSD. This observation had also been made by Snaphaan *et al*.^42^. According to several systematic reviews, patients with cognitive impairment are four times more likely to develop depression^43,44^. As well as TIA and minor stroke, the severity of a stroke measured by the NIHSS score was also a predictor of PSD. The relationship between the severity of the stroke and the occurrence of PSD^42,45,46^ and dementia^47^ has also been previously demonstrated. Diabetes mellitus also emerged as another predictor of PSD. Although there is not much evidence to support it, this is a finding that has been corroborated by Altieri *et al*.^3^ and Terroni *et al*.^43^.

The relationship between personal psychiatric history and the development of PSD has been supported by numerous studies and systematic reviews^48^. There is a greater risk of depression in patients who have previously suffered psychiatric disease.

It was surprising to note, however, that hyperintensities were only apparent in deep white-matter substance scores in the bivariate analysis of PSD carried out after 12 months. No other neuroimaging variable was associated with the development of PSD at baseline. Even so,in previous studies, the presence of depressive symptoms had been associated with the presence of acute ischemic lesions and/or cerebral microbleeds^19,49^.

Having suffered a previous stroke was related to PSA. Although there is not much evidence to support this, one previous study, by Yang *et al*., had reached the same conclusion^50^. After a stroke, a previous history of having had a stroke is a risk factor based on damage to strategic circuits, such as the frontal-subcortical circuit. We found an association between the presence of acute ischemic lesions in the basal ganglia and the Fazekas periventricular scale score in patients with basal apathy. Cortical atrophy has previously been strongly associated with symptoms of apathy^41^ and also with a reduction in white-matter integrity^51,52^ or white-matter hyperintensities located in the right hemisphere^40^. According to recent systematic reviews, the association between PSA and the location and/or size of the lesion cannot yet be clearly established^9,53^.

Similarly to with PSD, and in line with previous literature^11,12,33,38,51,54^, the persistence of apathy was associated with global cognitive impairment. The level of education was another factor that predicted apathy. Patients with only primary or lower levels of education faced a greater risk of suffering persistent apathy at 12 months after stroke than those with higher levels of education. Caeiro *et al*. had previously reported a similar relationship^55^.

Unlike its relationship with cognitive impairment and dementia, the role that alcohol plays in the development of apathy has previously not received very much academic attention. Numerous studies have analysed the effects of alcohol consumption on cognitive performance, but this still remains a controversial issue^56,57^. Considering the fact that the relationship between apathy and cognitive deterioration has now been demonstrated, it is possible to hypothesize that the effect of alcohol on apathy could be mediated by the effect that its consumption has on cognitive performance and that, in turn, this may affect the appearance of symptoms of apathy.

The limitations of our research that should be highlighted include the size of the cohort used, which limited the possibility of generalizing our results. In addition, the small sample limited the possibility of conducting an exhaustive study of the correlation between the different anatomical regions and the development of PSA or PSD. Moreover, no sample size calculation was performed. In order to make a complete evaluation, we performed several different tests. This increased the number of variables in the statistical analysis and consequently made some of the interpretations more difficult. This may have also increased the risk of type I error due to multiple testing. The excess of analysed variables in contrast to the small sample size could have also affected results of the regression analysis. An important limitation was the fact that we did not analyse the effect of antidepressant treatment. Finally, it should be added that we did not include a control group. This limited our ability to determine whether PSD or PSA was directly related to the ischemic event, or if this was a reactive phenomenon.

In conclusion, our research is one of the few longitudinal studies to have exclusively focused on depression and apathy after TIA and minor stroke. Interestingly, although individuals with TIA do not have any persistent functional deficits after stroke, they do have neuropsychiatric complaints such as PSA and PSD, which deserve clinical attention. Our findings have allowed us to extend the existing scientific evidence about the risk factors associated with the development of PSD and PSA. Depression appeared to be relatively high at the acute stage after TIA and minor stroke but, in contrast to apathy, to generally decrease during the follow-up period. Further research is needed to establish the optimal management procedure to help prevent the development of these neuropsychiatric complications. New research should be focus in the explanation and treatment of the persistent PSA that emerged as the most clinical relevant affective impairment in these patients.

## Supplementary information

Online supplementary information
