# Supplementary material for: Depression and Apathy After Transient Ischemic Attack or Minor Stroke: Prevalence, Evolution and Predictors
Source: Sci Rep. 2019 Nov 7;9:16248. doi: 10.1038/s41598-019-52721-5 (PMC6838079; doi:10.1038/s41598-019-52721-5)
Supplement: Supplementary file 1 — Online supplementary information [file 41598_2019_52721_MOESM1_ESM.doc]

**SUPPLEMENTARY INFORMATION**

**Title of the manuscript:**

DEPRESSION AND APATHY AFTER TIA OR MINOR STROKE: PREVALENCE, EVOLUTION AND PREDICTORS

**Author and co-author details**:

**Anna CARNES-VENDRELL**.

PhD. Clinical Psychologist and Neuropsychologist. Neurology department. Hospital Universitari de Santa Maria Clinical Neuroscience Group of the Biomedical Research Institute of Lleida (IRBLleida). Universitat de Lleida.

Avda Rovira Roure, 80. Lleida 25198, Spain.

**Joan DEUS**.

PhD. University of Barcelona, Spain. MRI Research Unit, Department of Radiology, Hospital del Mar, Barcelona, Spain. Department of Clinical and Health Psychology, Faculty of Psychology at the Autonomous University of Barcelona, Spain.

**Jessica MOLINA-SEGUIN**.

MD. Neurologist at Neurology Service Hospital Universitari Mutua de Terrassa. Clinical Neuroscience Group of the Biomedical Research Institute of Lleida (IRBLleida). Universitat de Lleida.

Avda Rovira Roure, 80. Lleida 25198, Spain.

**Josep PIFARRÉ**.

PhD, MD. Psychiatrist. Biomedical Research Institute of Lleida (IRBLleida) Universitat de Lleida.

Avda Rovira Roure, 80. Lleida 25198, Spain.

*** Francisco PURROY**.

PhD, MD. Stroke Unit. Hospital Universitari Arnau de Vilanova. Clinical Neuroscience Group of the Biomedical Research Institute of Lleida (IRBLleida). Universitat de Lleida.

Avda Rovira Roure, 80. Lleida 25198, Spain.

fpurroygarcia@gmail.com

Neuroimaging data:

44 (59.5%) of the patients had positive diffusion-weighted imaging. Most of the acute ischemic lesions affected the cortical MCA territory (28.4%). Cerebral microbleeds were detected in 10 (13.5%) patients. 21 (28.8%) patients had suffered chronic infarcts. Subcortical and periventricular leukoaraiosis levels of greater than one were observed in 17 (23.0%) and 29 (39.2%) patients, respectively.

Online supplementary table 1: Neuroimaging data

| **Variables** | **Total**  **n=74** |
| --- | --- |
| DWI positive | 44 (59.5) |
| DWI pattern   - Negative - SPOT - Cortical - Subcortical - Multiple | 32 (40.5)  15 (20.3)  13 (17.6)  16 (21.6)  0 (0) |
| Volume of acute infarctions.  Median (IR) cc | 0.87  (0.23-3.0) |
| **Acute ischemic lesions** | |
| Frontal lobe | 5 (6.8) |
| Temporal lobe | 9 (12.2) |
| Parietal lobe | 9 (12.2) |
| Occipital love | 1 (1.4) |
| Thalamus | 3 (4.1) |
| Basal ganglia | 4 (5.4) |
| Subcortical white matter lesion | 4 (5.4) |
| Brainstem | 2 (2.7) |
| Cerebellum | 1 (1.4) |
| ACA territory | 3 (4.1) |
| Cortical territory MCA | 21 (28.4) |
| Subcortical territory MCA | 9 (12.2) |
| PCA territory | 1 (1.4) |
| Penetrating arteries | 11 (14.9) |
| **CMBs** | |
| CMBs | 10 (13.5) |
| Lobe CMBs | 5 (6.8) |
| Deep CMBs | 8 (10.8) |
| **Chronic ischemic lesions** | |
| Fazekas PVL | 1.0 (0-2.0) |
| Fazekas HDWMS | 1.0 (0 -1.0) |
| Deep Chronic Infarctions | 13 (17.6) |
| Cortical Chronic Infarctions | 10 (13.5) |

Mean (SD) or median (IR) for quantitative variables. Frequencies (%) for qualitative variables.

Abbreviations: SPOT: scattered pearls in one arterial territory; ACA: anterior cerebral artery; MCA: medial cerebral artery; PCA: posterior cerebral artery; CMBs: cerebral microbleeds; PVL: Periventricular; HDWMS: Hyperintensities in the Deep White Matter Substance.

**Online supplementary table 2: Sociodemographic, clinical, cognitive and neuroimaging variables related to post-stroke depression and post-stroke apathy (baseline data)**

|  | **Post-stroke depression (PSD)** | | | **Post-stroke apathy (PSA)** | | |
| --- | --- | --- | --- | --- | --- | --- |
|  | **Healthy subjects**  **N=53** | **PSD subjects N=29** | ***p*** | **Healthy subjects**  **N=46** | **PSA subjects N=36** | ***p*** |
| **Affective variables** | | | | | | |
| **Barthel Index** | 100.0 [95.0-100.0] | 100.0 [85.0-100.0] | 0.093 | 100,0 (95,0-100,0) | 97,5 (88,8-100,0) | 0,092 |
| **mRS** | 1.0 [0.0-1.0] | 1.0 [1.0-2.0] | 0.081 | 1,0 (0,0-1,0) | 1,0 (1,0-2,0) | 0,188 |
| **AES** | 33.0 [27.0-39.0] | 40.0 [29.0-46.0] | **0.046** | 29.0 [25.0-33.0] | 43.5 [39.2-47.0] | **<0.001*** |
| **BDI-II** | 4.0 [2.0-6.0] | 9.0 [6.0-14.0] | **<0.001*** | 4,0 (2,0-7,5) | 6,0 (3,8-11,0) | **0,013*** |
| **MADRS** | 3.0 [2.0-5.0] | 9.0 [4.0-11.0] | **<0.001*** | 3,0 (1,2-5,0) | 7,0 (3,8-11,0) | **0,002*** |
| **ECVI-38** | 7.2 [3.9-12.5] | 13.8 [9.2-20.4] | **0.001*** | 9,5 (5,4-12,5) | 12,8 (6,2-19,7) | **0,047** |
| **Cognitive variables** | | | | | | |
| **MMSE** | 28.0 [26.0-29.0] | 26.0 [24.0-28.0] | **0.005*** | 28,0 (25,0-29,0) | 27,0 (26,0-29,0) | 0,565 |
| **MoCA** | 24.0 [22.0-27.0] | 25.0 [19.0-26.0] | 0.552 | 24,0 (21,0-26,0) | 25,0 (23,0-27,0) | 0,412 |
| **IQ level** | 92.4 (10.3) | 90.0 (8.5) | 0.286 | 93,3 (9,8) | 89,4 (9,2) | 0,075 |
| **Cognitive impairment:** |  | | 0.343 |  | | 0,129 |
| Mild cognitive impairment (0-2) | 31 (66.0%) | 14 (51.9%) |  | 28 (70,0) | 17 (50,0) |  |
| Moderate cognitive impairment (3+) | 16 (34.0%) | 13 (48.1%) |  | 12 (30,0) | 17 (50,0) |  |
| **Neuroimaging variables** | | | |  | | |
| **DWI diffusion:** |  | | 0.708 |  | | 0,849 |
| DWI negative | 19 (37.3%) | 12 (44.4%) |  | 18 (41,9) | 13 (37,1) |  |
| DWI positive | 32 (62.7%) | 15 (55.6%) |  | 25 (58,1) | 22 (62,9) |  |
| **DWI classification:** |  | | 0.948 |  | | 0,462 |
| Normal DWI | 19 (37.3%) | 12 (44.4%) |  | 18 (41,9) | 13 (37,1) |  |
| Scattered | 11 (21.6%) | 5 (18.5%) |  | 6 (14,0) | 10 (28,6) |  |
| Cortical | 10 (19.6%) | 4 (14.8%) |  | 9 (20,9) | 5 (14,3) |  |
| Subcortical | 11 (21.6%) | 6 (22.2%) |  | 10 (23,3) | 7 (20,0) |  |
| **Basal ganglia lesion** | 3 (5,9) | 1 (3,7) | >0.99 | 0 (0,0) | 4 (11,4) | **0,037** |
| **Total DWI volume** | 0.7 [0.2-2.1] | 1.0 [0.3-3.5] | 0.446 | 0,8 (0,2-3,0) | 0,8 (0,2-2,3) | 0,835 |
| **Presence of CMBs** | 6 (11.8%) | 4 (14.8%) | 0.731 | 5 (11,6) | 5 (14,3) | 0,746 |
| **Presence of lobar CMBs** | 4 (7.8%) | 1 (3.7%) | 0.654 | 3 (7,0) | 2 (5,7) | >0.99 |
| **Presence of deep CMBs** | 5 (9.8%) | 3 (11.1%) | 1.000 | 3 (7,0) | 5 (14,3) | 0,456 |
| **Fazekas PLV score** |  | | 0,343 |  | | **0,049** |
| 0 | 20 (37,7) | 16 (55,2) |  | 25 (54,3) | 11 (30,6) |  |
| 1 | 13 (24,5) | 3 (10,3) |  | 5 (10,9) | 11 (30,6) |  |
| 2 | 10 (18,9) | 4 (13,8) |  | 6 (13,0) | 8 (22,2) |  |
| 3 | 10 (18,9) | 6 (20,7) |  | 10 (21,7) | 6 (16,7) |  |
| **Fazekas HDWMS score** |  | | >0.99 |  | | 0,615 |
| 0 | 23 (43,4) | 12 (41,4) |  | 21 (45,7) | 14 (38,9) |  |
| 1 | 19 (35,8) | 11 (37,9) |  | 14 (30,4) | 16 (44,4) |  |
| 2 | 8 (15,1) | 5 (17,2) |  | 8 (17,4) | 5 (13,9) |  |
| 3 | 3 (5,7) | 1 (3,4) |  | 3 (6,5) | 1 (2,8) |  |
| **Chronic deep stroke** | 12 (23,5) | 2 (7,4) | 0,120 | 9 (20,9) | 5 (14,3) | 0,643 |
| **Chronic cortical stroke** | 7 (14,0) | 4 (15,4) | >0.99 | 5 (11,9) | 6 (17,6) | 0,527 |

Mean (SD) or median (IR) for quantitative variables. Frequencies (%) for qualitative variables.

Abbreviations: NIHSS: National Institute of Health Stroke Scale; mRS: Modified Rankin Scale; AES-C: Apathy Evaluation Scale, clinician version; BDI-II: Beck Depression Inventory; MADRS: Montgomery-Äsberg Depression Rating Scale; ECVI-38: Quality of Life Scale for Stroke; MMSE: Mini Mental State Examination; MoCA: Montreal Cognitive Assessment; IQ: intelligence quotient; DWI: diffusion-weighted magnetic resonance imaging; CMBs: cerebral microbleeds; PVL: Periventricular; HDWMS: Hyperintensities in the Deep White Matter Substance.

**Online supplementary table 3: Sociodemographic, clinical, cognitive and neuroimaging variables related to post-stroke depression and post-stroke apathy (12 months follow-up)**

|  | **Post-stroke depression (PSD)** | | | **Post-stroke apathy (PSA)** | | |
| --- | --- | --- | --- | --- | --- | --- |
|  | **Healthy subjects**  **N=62** | **PSD subjects N=8** | ***p*** | **Healthy subjects**  **N=45** | **PSA subjects N=25** | ***p*** |
| **Sociodemographic and clinical variables** | | | | | | |
| **Age** | 65,9 (11,3) | 63,2 (9,1) | 0,474 | 0,325 | 67,3 (10,4) | 0,325 |
| **Gender:** |  | | >0.99 |  | | 0,471 |
| Male | 45 (72,6) | 6 (75,0) |  | 31 (68,9) | 20 (80,0) |  |
| Female | 17 (27,4) | 2 (25,0) |  | 14 (31,1) | 5 (20,0) |  |
| **Family status:** |  | | 0,426 |  | | 0,944 |
| Married | 48 (77,4) | 7 (87,5) |  | 36 (80,0) | 19 (76,0) |  |
| Widower | 10 (16,1) | 0 (0,0) |  | 6 (13,3) | 4 (16,0) |  |
| Divorced / separated | 2 (3,2) | 1 (12,5) |  | 2 (4,4) | 1 (4,0) |  |
| Single | 2 (3,2) | 0 (0,0) |  | 1 (2,2) | 1 (4,0) |  |
| **Employment status:** |  | | **0,013*** |  | | **0,006*** |
| Employee | 19 (30,6) | 1 (12,5) |  | 16 (35,6) | 4 (16,0) |  |
| Unemployed | 3 (4,8) | 1 (12,5) |  | 2 (4,4) | 2 (8,0) |  |
| Retired | 38 (61,3) | 3 (37,5) |  | 27 (60,0) | 14 (56,0) |  |
| Inability to work | 2 (3,2) | 3 (37,5) |  | 0 (0,0) | 5 (20,0) |  |
| **Level of education:** |  | | 0,700 |  | | 0,071 |
| Primary education | 43 (69,4) | 5 (62,5) |  | 27 (60,0) | 21 (84,0) |  |
| Secondary education or more | 19 (30,6) | 3 (37,5) |  | 18 (40,0) | 4 (16,0) |  |
| **Occasional alcohol consume** | 28 (45,2) | 2 (25,0) | 0,452 | 19 (42,2) | 11 (44,0) | >0.99 |
| **Smoking:** |  | | 0,638 |  | | 0,983 |
| Non smoking | 27 (43,5) | 2 (25,0) |  | 19 (42,2) | 10 (40,0) |  |
| Ex-smoking | 19 (30,6) | 3 (37,5) |  | 14 (31,1) | 8 (32,0) |  |
| Smoking | 16 (25,8) | 3 (37,5) |  | 12 (26,7) | 7 (28,0) |  |
| **Diabetes mellitus** | 20 (32,3) | 6 (75,0) | **0,045*** | 12 (26,7) | 14 (56,0) | **0,030*** |
| **Hypertension** | 41 (66,1) | 4 (50,0) | 0,443 | 30 (66,7) | 15 (60,0) | 0,766 |
| **Hypercholesterolemia** | 25 (40,3) | 5 (62,5) | 0,275 | 18 (40,0) | 12 (48,0) | 0,692 |
| **Atrial fibrillation** | 3 (4,8) | 0 (0,0) | >0.99 | 2 (4,4) | 1 (4,0) | >0.99 |
| **Previous psychiatric disease** | 20 (32,3) | 4 (50,0) | 0,432 | 14 (31,1) | 10 (40,0) | 0,626 |
| **Family history:** |  | | 0,172 |  |  | 0,456 |
| Neurological disease | 25 (40,3) | 1 (12,5) |  | 19 (42,2) | 7 (28,0) |  |
| Psychiatric disease | 6 (9,7) | 2 (25,0) |  | 4 (8,9) | 4 (16,0) |  |
| **NIHSS score at admission** | 1,0 (0,0-2,0) | 3,0 (2,0-3,0) | **0,011*** | 1,0 (0,0-2,0) | 2,0 (0,0-3,0) | 0,091 |
| **Previous stroke** | 8 (12,9) | 3 (37,5) | 0,105 | 6 (13,3) | 5 (20,0) | 0,506 |
| **Etiology of stroke^1^:** |  | | **0,017*** |  | | 0,873 |
| Large artery atherosclerotic | 12 (19,4) | 4 (50,0) |  | 10 (22,2) | 6 (24,0) |  |
| Cardioembolism | 7 (11,3) | 2 (25,0) |  | 5 (11,1) | 4 (16,0) |  |
| Lacunar | 14 (22,6) | 2 (25,0) |  | 10 (22,2) | 6 (24,0) |  |
| Undetermined | 29 (46,8) | 0 (0,0) |  | 20 (44,4) | 9 (36,0) |  |
| **Affective variables** | | | | | | |
| **Barthel Index baseline** | 100,0 (95,0-100,0) | 95,0 (85,0-100,0) | 0,321 | 100,0 (95,0-100,0) | 95,0 (85,0-100,0) | **0,034*** |
| **mRS baseline** | 1,0 (0,0-1,0) | 1,0 (1,0-2,0) | 0,204 | 1,0 (0,0-1,0) | 1,0 (1,0-2,0) | **0,024*** |
| **AES-C baseline** | 33,7 (7,8) | 45,2 (10,6) | **0,018*** | 31,0 (6,1) | 42,4 (8,5) | **<0,001*** |
| **BDI-II baseline** | 4,5 (3,0-7,8) | 7,0 (2,8-13,2) | 0,404 | 4,0 (2,0-7,0) | 6,0 (4,0-9,0) | **0,043*** |
| **MADRS baseline** | 4,0 (2,0-7,0) | 6,5 (1,8-11,2) | 0,441 | 4,0 (2,0-7,0) | 5,0 (3,0-9,0) | 0,093 |
| **ECVI-38 baseline** | 9,5 (5,3-14,1) | 13,8 (9,0-19,1) | 0,123 | 7,2 (5,3-12,5) | 11,8 (9,2-18,4) | **0,011*** |
| **Cognitive variables** | | | | | | |
| **MMSE baseline** | 27,0 (25,0-29,0) | 27,5 (22,8-29,0) | 0,696 | 28,0 (25,0-29,0) | 26,0 (24,0-28,0) | 0,064 |
| **MoCA baseline** | 24,0 (21,2-26,0) | 26,5 (23,2-28,2) | 0,201 | 24,0 (22,0-26,0) | 24,0 (21,0-25,0) | 0,694 |
| **IQ level baseline** | 91,7 (9,3) | 92,1 (13,3) | 0,931 | 92,9 (9,1) | 89,6 (10,6) | 0,201 |
| **Cognitive impairment:** |  | | 0,124 |  | | 0,063 |
| Mild cognitive impairment (0-2) | 42 (67,7) | 3 (37,5) |  | 33 (73,3) | 12 (48,0) |  |
| Moderate cognitive impairment (3+) | 20 (32,3) | 5 (62,5) |  | 12 (26,7) | 13 (52,0) |  |
| **Neuroimaging variables** | | | |  | | |
| **DWI diffusion:** |  | | 0,451 |  | | 0,489 |
| DWI negative | 28 (45,9) | 2 (25,0) |  | 21 (47,7) | 9 (36,0) |  |
| DWI positive | 33 (54,1) | 6 (75,0) |  | 23 (52,3) | 16 (64,0) |  |
| **DWI classification:** |  | | 0,482 |  | | 0,136 |
| Normal DWI | 28 (45,9) | 2 (25,0) |  | 21 (47,7) | 9 (36,0) |  |
| Scattered | 10 (16,4) | 3 (37,5) |  | 5 (11,4) | 8 (32,0) |  |
| Cortical | 10 (16,4) | 1 (12,5) |  | 9 (20,5) | 2 (8,0) |  |
| Subcortical | 13 (21,3) | 2 (25,0) |  | 9 (20,5) | 6 (24,0) |  |
| **Basal ganglia lesion** | 2 (3,3) | 1 (12,5) | 0,313 | 2 (4,5) | 1 (4,0) | >0.99 |
| **Total DWI volume** | 0,7 (0,2-3,0) | 0,5 (0,1-1,0) | 0,251 | 0,7 (0,2-1,8) | 1,0 (0,3-3,0) | 0,502 |
| **Presence of CMBs** | 8 (13,1) | 1 (12,5) | >0.99 | 6 (13,6) | 3 (12,0) | >0.99 |
| **Presence of lobar CMBs** | 5 (8,2) | 0 (0,0) | >0.99 | 3 (6,8) | 2 (8,0) | >0.99 |
| **Presence of deep CMBs** | 6 (9,8) | 1 (12,5) | >0.99 | 4 (9,1) | 3 (12,0) | 0,698 |
| **Fazekas PLV score** |  | | 0,903 |  | | 0,126 |
| 0 | 29 (46,8) | 3 (37,5) |  | 25 (55,6) | 7 (28,0) |  |
| 1 | 9 (14,5) | 1 (12,5) |  | 6 (13,3) | 4 (16,0) |  |
| 2 | 11 (17,7) | 2 (25,0) |  | 6 (13,3) | 7 (28,0) |  |
| 3 | 13 (21,0) | 2 (25,0) |  | 8 (17,8) | 7 (28,0) |  |
| **Fazekas HDWMS score** |  | | **0,041*** |  | | 0,078 |
| 0 | 27 (43,5) | 0 (0,0) |  | 22 (48,9) | 5 (20,0) |  |
| 1 | 21 (33,9) | 6 (75,0) |  | 14 (31,1) | 13 (52,0) |  |
| 2 | 10 (16,1) | 2 (25,0) |  | 6 (13,3) | 6 (24,0) |  |
| 3 | 4 (6,5) | 0 (0,0) |  | 3 (6,7) | 1 (4,0) |  |
| **Chronic deep stroke** | 11 (18,0) | 1 (12,5) | >0.99 | 9 (20,5) | 3 (12,0) | 0,515 |
| **Chronic cortical stroke** | 8 (13,3) | 2 (28,6) | 0,279 | 3 (7,0) | 7 (29,2) | **0,028*** |

^1^ The stroke or TIA mechanism was determined using the results of a detailed assessment and were classified into etiologies based on the Trial of Org 10172 in Acute Stroke (TOAST) guidelines.

Mean (SD) or median (IR) for quantitative variables. Frequencies (%) for qualitative variables.

Abbreviations: NIHSS: National Institute of Health Stroke Scale; mRS: Modified Rankin Scale; AES-C: Apathy Evaluation Scale, clinician version; BDI-II: Beck Depression Inventory; MADRS: Montgomery-Äsberg Depression Rating Scale; ECVI-38: Quality of Life Scale for Stroke; MMSE: Mini Mental State Examination; MoCA: Montreal Cognitive Assessment; IQ: intelligence quotient; DWI: diffusion-weighted magnetic resonance imaging; CMBs: cerebral microbleeds; PVL: Periventricular; HDWMS: Hyperintensities in the Deep White Matter Substance.
